# Successful in situ replantation of fingertip amputation without vascular anastomosis: A case report

**DOI:** 10.1097/MD.0000000000049219

**Published:** 2026-06-05

**Authors:** Peng Sun, Xiaoming Cai, Xiaofeng Wang

**Affiliations:** a Department of Hand Microsurgery and Plastic Reconstructive Surgery, Ningbo No.6 Hospital, Ningbo, Zhejiang, China; b Ningbo Clinical Research Center for Orthopedics, Sports Medicine & Rehabilitation, Ningbo, Zhejiang, China.

**Keywords:** fingertip amputation, non-anastomotic technique, replantation, Tamai classification

## Abstract

**Rationale:**

Fingertip amputations at Tamai zone I, the most distal anatomical level, remain a reconstructive challenge. Although vascular anastomosis has been emphasized in replantations, viable outcomes may still be achieved without revascularization under specific conditions.

**Patient concerns:**

A 17-year-old female factory worker incurred a sharp oblique amputation of the distal left index finger due to a machine-related occupational injury. The amputation plane was located distal to the lunula, as confirmed by radiographic assessment, which demonstrated that less than one-third of the distal phalanx was involved.

**Diagnoses:**

Complete fingertip amputation classified as Tamai zone I, with no evidence of crush injury or contamination and a well-preserved distal stump.

**Interventions:**

In situ replantation was performed without vascular or nerve anastomosis, as the vessels and nerves were too small for reliable microsurgical repair. The distal phalanx was stabilized using a single K-wire. Postoperative management included strict bed rest, subcutaneous administration of low-molecular-weight heparin, and administration of prophylactic antibiotics.

**Outcomes:**

The replanted fingertip gradually regained perfusion without necrosis or infection. The patient was discharged uneventfully 1 week after surgery. By 12 weeks, the protective sensation had returned, nail growth was satisfactory, and radiographs confirmed the union of the distal phalanx.

**Lessons:**

Replantation without vascular anastomosis can achieve satisfactory functional and cosmetic outcomes in selected distal fingertip amputations, particularly in Tamai zone I. Careful patient selection is crucial, considering factors such as injury type, wound contamination, and patient age. This approach should be considered when feasible, especially in environments with limited medical resources or when vessel caliber precludes anastomosis.

## 1. Introduction

Fingertip amputation is one of the most frequently encountered injuries in hand trauma, particularly among young individuals and workers involved in manual tasks.^[1]^ The distal fingertip segment, especially in Tamai zone I, presents unique reconstructive challenges owing to its extremely small vascular caliber, with vessel diameters often <0.3 mm. It is widely believed that successful fingertip replantation is primarily dependent on precise vascular anastomosis; however, in cases of certain distal fingertip amputations, this approach can be technically challenging and may not be practical.^[2]^

In cases where microsurgical revascularization cannot be performed for distal fingertip amputations, non-vascularized composite grafting serves as an alternative treatment, although it is often accompanied by unpredictable graft survival, lack of skeletal support, and suboptimal cosmetic and sensory outcomes.^[3]^ The initial survival mechanism depends on plasmatic imbibition from the recipient bed, followed by gradual neovascularization.

This report describes an unusual case of in situ replantation following Tamai zone I amputation of the distal index finger, performed without vascular or neural anastomosis. The procedure involved in situ replantation using skeletal fixation with a Kirschner wire, which preserved the original structural integrity of the distal segment and resulted in complete graft survival with excellent functional outcomes. Although this approach shares some features with composite grafting, it differs in that the amputated segment is anatomically aligned and stabilized to preserve local microcirculation and promote faster revascularization. Early reperfusion and successful outcomes in such cases are rarely reported, making the present case noteworthy.

## 2. Case presentation

**Figure 1.**
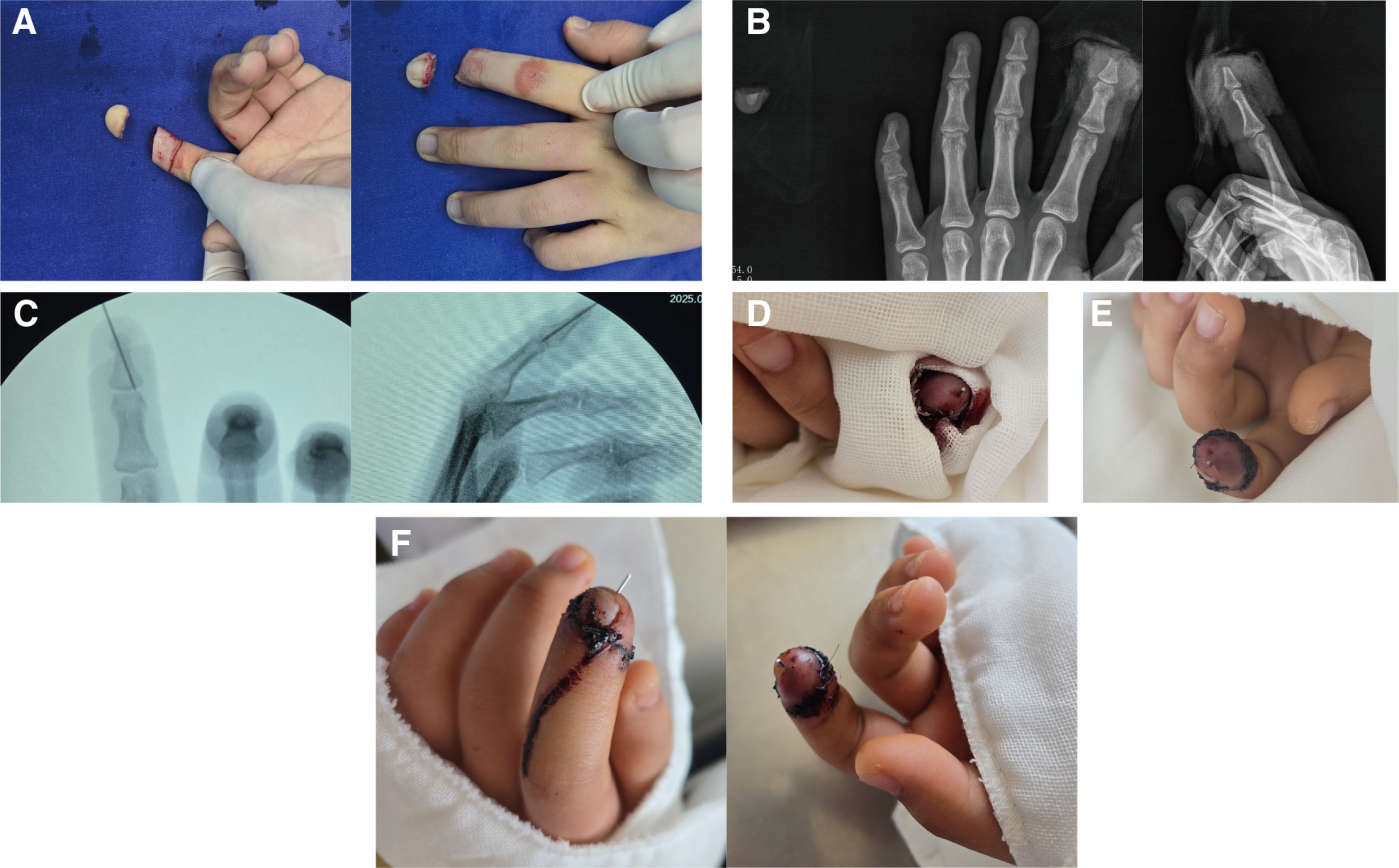
Preoperative photograph (A) and radiographs (B) of the left index finger showing an oblique amputation at the distal phalanx level (Tamai zone I). (C) Intraoperative radiograph demonstrating Kirschner-wire fixation. (D) Postoperative photograph taken at 2 hours demonstrating restoration of circulation in the replanted fingertip, with improved skin color and capillary refill. (E) Postoperative photograph taken at 3 days showing a purplish discoloration of the replanted fingertip with delayed capillary refill. (F) Postoperative photograph taken on postoperative day 7 (day of discharge) showing good circulation in the fingertip with normal color and brisk capillary refill.

A 17-year-old right-handed girl underwent transverse-oblique amputation of the distal segment of the left index finger, extending beyond the dorsal lunula, caused by a sharp industrial cutting machine. The injury was classified as Tamai zone I, involving less than one-third of the distal phalanx with a clean, sharply defined wound margin, as confirmed by radiographic assessment (X-ray; Fig. 1A and B). The amputated part was wrapped in saline-moistened gauze and stored at 4°C. Surgery was performed 4 hours after the injury, as the patient required transportation from a distant location to our hospital.

Minimal debridement of the amputated parts and digital stumps was performed under a brachial plexus block, limited to nonviable skin and subcutaneous tissue to preserve maximal viable structures, including the nail bed and distal pulp. Intraoperative exploration using an operating microscope revealed no identifiable vessels or nerves suitable for anastomosis. Only tiny distal branches of the digital artery were present, and no veins or nerves could be clearly identified. A single 0.8-mm Kirschner wire was inserted longitudinally across the fracture site to ensure stable bone fixation. The soft tissues were approximated with minimal sutures, and the nail plate was precisely realigned and fixed in situ (Fig. 1C), while the nail bed was left unsutured to avoid additional trauma and to preserve its delicate vascular supply, which facilitates proper nail regeneration. Only soft cotton padding was used to wrap the left hand, and no pressure dressing was applied to the graft.

Toward the end of the operation, the replanted segment remained pale, with no visible signs of perfusion or active bleeding. Interestingly, 2 hours postoperatively, the replanted fingertip appeared pink and well-perfused (Fig. 1D). Capillary refill and color were monitored in the ward at hourly intervals. Postoperatively, the patient was administered low-molecular-weight heparin 3200 IU subcutaneously once daily, papaverine 30 mg intramuscularly every 8 hours, and prophylactic antibiotics. Bed rest was maintained, and a heat lamp was used to warm the affected hand to promote vasodilation and enhance perfusion to the replanted fingertip. The first dressing change was performed on postoperative day 3, when the replanted fingertip appeared dusky with purplish discoloration and delayed capillary refill, possibly due to transient venous congestion (Fig. 1E). Close observation and warm protection were maintained. The symptoms gradually improved within 24 hours, and stable perfusion was confirmed thereafter. The patient was discharged on postoperative day 7 with a well-perfused fingertip, normal coloration, capillary refill, and no postoperative complications (Fig. 1F).

**Figure 2.**
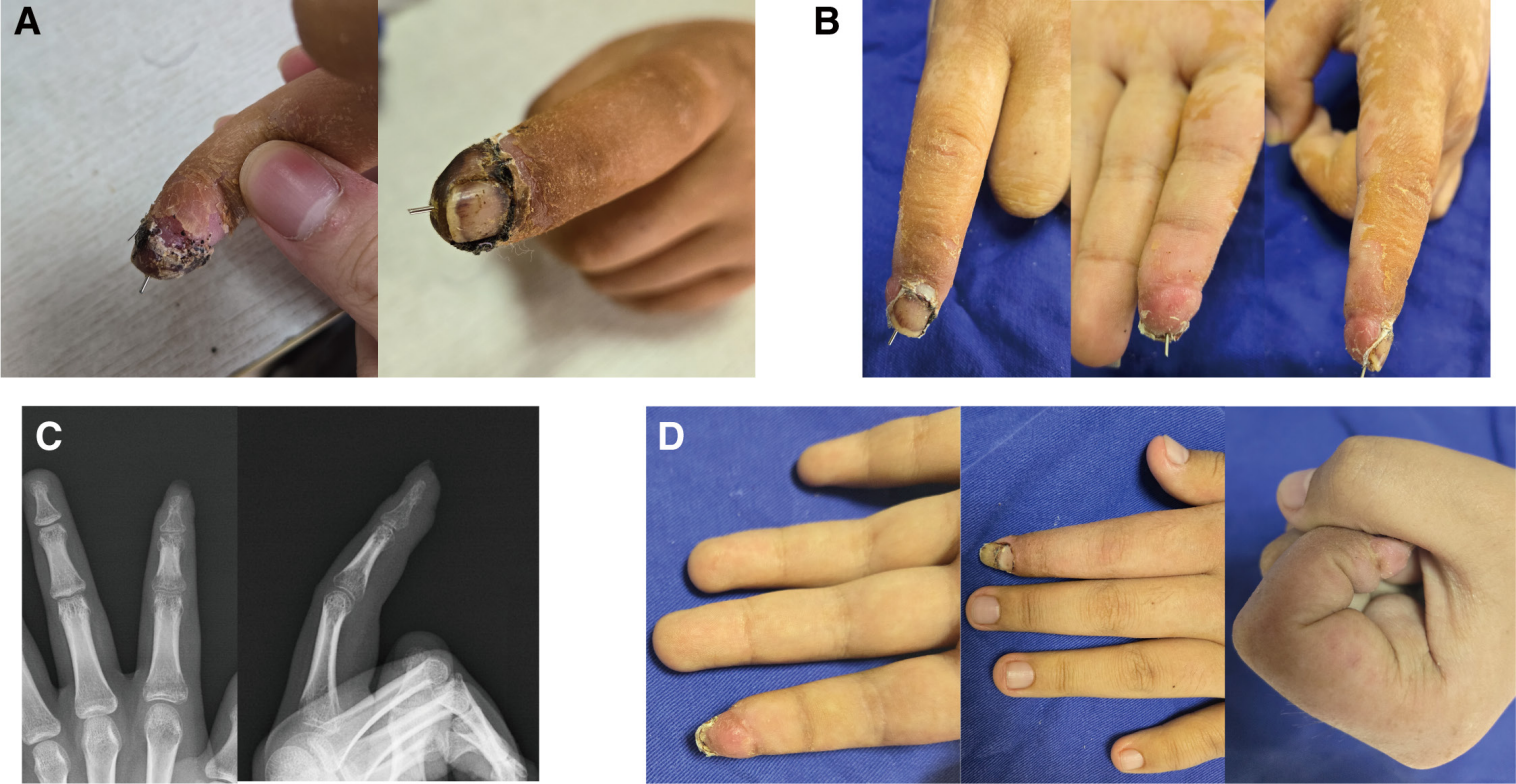
Postoperative photographs after 4 weeks (A) and 8 weeks (B). At 12 weeks postoperatively, radiograph (C) demonstrating complete bony union of the index finger and functional recovery (D) of the replanted fingertip.

At the 4-week postoperative follow-up, the replanted fingertip exhibited gradual desquamation, with complete tissue survival (Fig. 2A). As the patient returned to her hometown, no outpatient follow-up was performed until approximately 12 weeks postoperatively, when the Kirschner wire had been retained for 12 weeks and was subsequently removed. By that time, the replanted fingertip had begun to show signs of nail growth, and protective sensation had returned. However, fine discriminatory function remained coarse, with 2-point discrimination measuring >15 mm. Radiographs demonstrated satisfactory bone healing, and the patient’s finger mobility was nearly normal. (Fig. 2C and D) The patient expressed satisfaction with both the cosmetic appearance and functional recovery of the finger, reporting no pain but a slightly reduced sensation.

At 6 months postoperatively, the patient was invited for an additional functional assessment using the Disabilities of the Arm, Shoulder and Hand questionnaire to provide an objective reference. The Disabilities of the Arm, Shoulder and Hand score was 2, indicating minimal disability and consistent with the satisfactory recovery observed at 3 months after surgery.

## 3. Discussion

Fingertip replantation by restoring native tissue is considered the ideal treatment for achieving optimal aesthetic and functional outcomes, preserving digital length, maintaining a painless and sensate fingertip, ensuring durable soft-tissue coverage, and retaining the nail.^[4]^ Conventionally, fingertip replantation relies on meticulous microvascular repair to maximize survival and functional recovery. However, in Tamai zone I injuries, the diminutive caliber of the digital arteries and veins often renders vascular anastomosis technically challenging or impossible. As a result, alternative approaches, such as composite grafting or non-anastomotic replantation, have been explored, albeit with variable success.^[5,6]^

Early studies – particularly in pediatric and adult populations – have suggested that composite graft survival may rely on a series of physiological processes: plasmatic imbibition, capillary inosculation, and subsequent neovascularization, enabling distal tissue segments to survive despite the absence of direct arterial or venous anastomosis.^[7,8]^ These mechanisms are thought to be most effective in well-vascularized wound beds with small-volume amputations and prompt surgical intervention.

Our case presents a mechanism markedly different from the scenarios outlined above. In conventional non-vascularized composite grafts, the primary goal is to cover the wound and restore appearance, with fixation usually limited to soft-tissue suturing. Tissue survival mainly depends on passive diffusion from the recipient bed until neovascularization occurs, which often leads to partial necrosis when the graft is large.

In contrast, our in situ replantation preserved the anatomical alignment and residual microcirculatory connections between the stump and the amputated part. Stable Kirschner-wire fixation ensured accurate alignment and stability of the replanted segment. Postoperative management also differed from that of conventional composite grafts. The patient was kept on strict bed rest with local warming using a heat lamp to promote circulation in the replanted fingertip. Low-molecular-weight heparin and papaverine were administered to prevent thrombosis and improve microvascular perfusion. These measures are rarely applied in simple composite grafts.

Although vascular anastomosis was not performed, the procedure more closely resembled replantation rather than a conventional non-vascularized composite graft. Unlike most conventional composite grafts, no pressure dressing was applied to the affected finger, allowing real-time monitoring of vascular changes. Although the replanted finger appeared pale immediately after surgery, perfusion was restored within 2 hours postoperatively. While mild cyanosis was observed on postoperative day 3, by discharge on day 7, the fingertip circulation had become robust and stable. Without arterial anastomosis, the replanted fingertip did not become tense at any point during the postoperative period. It stayed supple, and there was no need for any measures, such as needling or decompression. This may be attributed to the limited arterial inflow, which kept venous outflow pressure low. Postoperative low-molecular-weight heparin may help prevent small blood clots and maintain adequate perfusion of the fingertip, even without vascular anastomosis.

In this case, nerve repair was not performed because the distal nerve stumps were too small for reliable microsurgical repair. In fingertip replantation, nerve repair is not regarded as a routine procedure because favorable sensory recovery can occur even without nerve suturing.^[9,10]^ Consistent with these findings, our patient demonstrated partial protective sensation and coarse 2-point discrimination at 12 weeks postoperatively. The patient’s young age (17 years) likely facilitated rapid tissue regeneration and contributed to the excellent functional and cosmetic outcome.

In previously reported pediatric cases of distal fingertip amputation, complete survival of composite grafts was only about 17%, increasing to 81% when partial graft take was included.^[3]^ In our case, the replanted fingertip survived completely, with rapid restoration of perfusion. Functional recovery and cosmetic appearance were excellent, highlighting the favorable outcome achievable in carefully selected Tamai zone I injuries.

The patient’s injury, characterized by clean-cut amputation and rapid surgical intervention, likely contributed to the favorable outcome.^[11,12]^ Stable bone fixation, tension-free soft-tissue closure, and maximal preservation of viable tissue structures are critical for maintaining tissue viability in the absence of revascularization.

This technique offers several advantages in practice. By eliminating microvascular repair, the procedure becomes simpler and faster, with fewer complications. It is especially helpful in facilities without microsurgery capabilities or in cases where vessel quality is poor.

However, this technique is not suitable in all situations. It appears to work best for patients who come early with tidy, non-contaminated cuts, small distal defects, and youth. If the injury involves crushing, avulsion, heavily contaminated wounds, advanced age, significant soft tissue or bone damage, or a significant delay before surgery, the outcome may be much less favorable.^[7]^ Ideal candidates for in situ replantation without vascular anastomosis include patients with clean-cut injuries, short ischemia time, younger age, and amputations limited to Tamai zone I. Potential pitfalls of this technique include infection, delayed healing, and nail deformities, although none occurred in this case. In resource-limited settings, the success of this approach may be limited by the ability to closely monitor postoperative perfusion and manage complications effectively. In crush injuries, modifications such as careful debridement, staged reconstruction, or adjunctive flap coverage may be required to improve outcomes.

Further investigation is needed to clarify appropriate patient selection, enhance surgical protocols, and evaluate long-term functional outcomes. Collaboration across institutions and the inclusion of broader patient samples may yield stronger data to support clinical decision-making.

## 4. Conclusion

In the present case of Tamai zone I fingertip amputation, replantation without vascular anastomosis resulted in satisfactory survival and functional recovery, suggesting that it may be a feasible option for carefully selected patients. Achieving the best results depends on selecting the right patient, promptly performing surgery, and ensuring stable bone fixation. This simpler method may be especially useful when microsurgical repair is not an option or when medical resources are limited. However, further research is necessary to develop clear guidelines and assess their long-term effectiveness.

## Acknowledgments

The authors thank the patient’s contribution and the guardian’s written permission to publish this case report.

## Author contributions

**Conceptualization:** Peng Sun, Xiaoming Cai.

**Data curation:** Peng Sun.

**Formal analysis:** Peng Sun, Xiaoming Cai.

**Writing – original draft:** Peng Sun.

**Writing – review & editing:** Peng Sun, Xiaofeng Wang.
